# Is preoperative serum lactate dehydrogenase useful in predicting the outcomes of patients with upper tract urothelial carcinoma?

**DOI:** 10.1002/cam4.1751

**Published:** 2018-08-27

**Authors:** Ping Tan, Jie Chen, Nan Xie, Hang Xu, Jianzhong Ai, Huan Xu, Liangren Liu, Lu Yang, Qiang Wei

**Affiliations:** ^1^ Department of Urology West China Hospital Sichuan University Chengdu China; ^2^ Institute of Urology West China Hospital Sichuan University Chengdu China; ^3^ Department of Radiology West China Hospital Sichuan University Chengdu China; ^4^ Department of Emergency West China Hospital Sichuan University Chengdu China; ^5^ Department of Pathology West China Hospital Sichuan University Chengdu China

**Keywords:** biomarkers, lactate dehydrogenase, radical nephroureterectomy, upper urinary tract, urothelial carcinoma

## Abstract

**Background:**

Lactate dehydrogenase (LDH) has been proved to be associated with clinical outcomes in various carcinomas; however, limited evidence was available in upper urinary tract urothelial carcinoma (UTUC). Thus, the aim of this study was to evaluate the prognostic impact of LDH in UTUC.

**Patients and methods:**

A cohort of 668 patients WERE retrospectively included between 2003 and 2016. Kaplan‐Meier method and Cox proportional hazards regression models were used to evaluate the association of LDH with overall survival (OS), cancer‐specific survival (CSS), disease recurrence‐free survival (RFS), and metastasis‐free survival (MFS). The cutoff level of LDH was set at 220 U/L for the upper limit of normal.

**Results:**

Kaplan‐Meier plots showed the group with elevated LDH had significant poor OS (*P* = 0.003), CSS (*P* = 0.005), and RFS (*P* = 0.005), but not MFS (*P* = 0.099). However, multivariate Cox analysis suggested that LDH was not an independent predictor for CSS (HR 1.50, 95%CI: 0.87‐2.59), OS (HR 1.56, 95%CI: 0.94‐2.58), RFS (HR 1.33, 95%CI: 0.83‐2.12), or MFS (HR 1.16, 95%CI: 0.79‐1.71). Albumin, globulin, and HBDH were also not related to survival outcomes of UTUC patients in multivariate analysis, while higher alkaline phosphatase was associated with worse CSS and OS, and higher white blood cells contributed to poor CSS and RFS. In subgroup analysis, results found higher LDH was associated with poor OS in patients with localized disease (pT ≤ 2) (HR 4.03, 95%CI: 1.37‐11.88).

**Conclusion:**

The preoperative LDH was not an independent prognostic factor for patients with UTUC, while elevated LDH was proved to be correlated with worse OS in patients with localized disease.

## 1 INTRODUCTION

Upper tract urothelial carcinoma (UTUC), including renal pelvicalyceal and ureteric urothelial carcinoma, accounts for approximately 5% of urothelial carcinomas and is typically accompanied by multiple lesions, high recurrence rates, and distant metastasis.^1^, ^2^ The UTUC incidence rate is approximately 0.2% in Western countries but that is higher in Asian countries because of Chinese herds and arsenic exposure.^1^ Radical nephroureterectomy (RNU) with bladder cuff excision is still the reference standard treatment for UTUC to date.^1^ Despite the advancement of surgical techniques and benefits of neoadjuvant or adjuvant intervention, the survival outcomes of patients with UTUC have not been improved significantly over time. Thus, the identification of prognostic factors is of paramount importance to adapt treatment in time.

Previous studies have figured out the metabolism of cancer cells differs from that of normal cells. Even in the presence of adequate oxygen, cancer cells preferentially metabolize glucose by glycolysis to generate sufficient energy for proliferation and development, which is known as the Warburg effect and is one of the predominant metabolic alterations that occur during malignant transformation.^3^ Lactate dehydrogenase (LDH), which regulated by hypoxia‐inducible factor‐1 alpha (HIF‐1a), is involved in the glycolytic pathway and catalyzes the conversion of pyruvate and lactate coupled with the conversion of NADH and NAD+. Serum LDH level could reflect these metabolic changes.^4^

Elevated LDH level has been incorporated into prognostic scores for several types of cancer, including renal cell carcinoma, melanoma, prostate cancer, lung cancer, and colorectal cancer.^5^ Recently, a study reported that preoperative serum LDH was an independent prognostic factor for patients with UTUC. However, their results may not be reliable due to only 100 patients were included with 10 cases had elevated serum LDH.^6^ Therefore, the aim of this study was to further evaluate the prognostic values of preoperative LDH in patients with UTUC after RNU treatment in our center.

## 2 PATIENTS AND METHODS

### 2.1 Patients

A total of 710 patients with UTUC received RNU with bladder cuff excision treatment between 2003 and 2016 in West China Hospital. The clinicopathological data including age, gender, anemia, perioperative blood transfusion, tumor side and location, size, tumor grade, TNM classification, lymph node status, surgical margin status, multifocality, concomitant variant histology (CVH), lymphovascular invasion (LVI), tumor architecture, and adjuvant therapy were collected. Patients with preoperative infection, liver diseases, fever, other tumors, with the previous cystectomy for invasive bladder cancer, or who received the treatment of neoadjuvant or adjuvant chemotherapy or radiotherapy before surgery were excluded. The LDH value and other serum biochemical patterns of each patient were extracted from the most recent routine examines within 1 month before surgery. Patients with missing LDH value were also excluded from our cohort. Lymph node dissection was not routinely performed. Eventually, 668 patients were enrolled in this retrospective study. The study was approved by the Ethics Committee of West China Hospital, and the methods were carried out in accordance with the approved guidelines. For this type of study, informed consent is not required.

### 2.2 Pathological evaluation

All RNU specimens were, respectively, re‐elevated by two separately specific pathologists according to standard procedures. The 2010 American Joint Committee of Cancer TNM classification and the WHO International Society of Urological Pathology consensus classification were used to evaluate the tumor stage and grade, respectively.

### 2.3 Cutoff value selection

The cutoff value of LDH was determined as the upper limit of normal range from West China Hospital, which was 220 U/L. Higher than the cutoff value was considered as a high level of LDH. Other serum biochemical markers including alpha‐hydroxybutyrate dehydrogenase (HBDH, >180 U/L vs ≤180 U/L), alkaline phosphatase (ALP, >90 vs ≤90 U/L), albumin (ALB, >35 vs ≤35 g/L), globulin (GLB, >30 vs ≤30 g/L), and white blood cells (WBC, >8.3 vs ≤8.3*10^9^/L) were also included in analysis.

### 2.4 Follow‐up strategies

Patients were followed every 3‐4 months for the first year after surgery according to the guideline, semiannually for the second and third year, and annually thereafter, or as clinically indicated with urinary cytology and excretory urography of the contralateral upper urinary tract, and routine check‐ups that included history, physical examination, blood laboratory tests, and chest radiography. If clinically indicated, selective bone scan and chest/abdomen CT/MRI were elevated.

Disease recurrence was defined as local recurrence in the operating field, lymph node spread and/or distant metastasis that had not been found in the preoperative examination. Specifically, the tumor found in the urinary bladder or contralateral upper urinary tract after surgery was not regarded as tumor relapse.

### 2.5 Statistical analysis

Continuous variables were analyzed using Student's *t* test, and categorical variables were elevated using the chi‐squared test or Fisher's exact test. Probabilities of overall survival (OS), cancer‐specific survival (CSS), disease recurrence‐free survival (RFS), and metastasis‐free survival (MFS) were estimated using the Kaplan‐Meier method, and the log‐rank test was used to assess differences. Univariate and multivariable Cox's proportional hazards regression models were used to evaluate the relationships between variables and OS, CSS, RFS, and MFS. Risk factors with a *P* value <0.15 in the univariate analysis were included in the multivariate analysis model. Hazard ratios (HRs) with their 95% CIs were used to assess the strength of the individual variables. All reported *P* values were two‐sided with statistical significance set at *P* < 0.05. Statistical analyses were performed using IBM SPSS Statistics version 22.0 (IBM Corp., Armonk, NY, USA).

## 3 RESULT

The clinicopathological features of the patient cohort included in this study are shown in Table 1. The mean age of patients was 65.8 ± 11.39 years old. The median follow‐up for the whole cohort was 45 (interquartile range [IQR]: 21‐74) months. The median serum LDH in the patients was 179 (IQR: 158‐204) U/L. Patients with LDH >220 U/L was significantly associated with gender, perioperative blood transfusion, and tumor grade (all *P* < 0.05). In particular, patients with LDH >220 U/L had a greater probability (84.0% vs 72.2%) of having a high‐grade disease and had more females (55.7% vs 40.7%) (Table 1).

**Table 1:** Baseline characteristics of patients with urinary tract urothelial carcinoma included in present study

Variables	Total (n = 668)	LDH > 220 U/L (n = 106)	LDH ≤ 220 U/L (n = 562)	*P*
Age (>67/≤67 y)	330/338	49/57	281/281	0.272
Gender (male/female)	380/288	47/59	333/229	0.003
Tumor side (left/right)	344/324	58/48	286/276	0.525
Perioperative blood transfusion (yes/no)	99/569	25/81	74/488	0.011
Anemia (yes/no)	269/399	49/57	220/342	0.195
Tumor location, n (%)				
Pelvicalyceal	353 (52.8)	64 (60.4)	289 (51.4)	0.134
Ureteric	196 (29.3)	23 (21.7)	173 (30.8)	
Both	119 (17.8)	19 (17.9)	100 (17.8)	
Tumor grade (high/low)	495/173	89/17	406/156	0.011
Tumor stage (≤pT2/≥pT3)	338/330	48/58	290/272	0.245
Lymph node status, n (%)				
pN0	80 (12.0)	13 (12.3)	67 (11.9)	0.130
pN+	64 (9.6)	16 (15.1)	48 (8.5)	
pNx	524 (78.4)	77 (72.6)	447 (79.5)	
LVI (with vs without)	99/569	16/90	83/479	0.883
Tumor size (>3 cm vs ≤3 cm)	450/218	75/31	375/187	0.498
Surgical margin status (positive vs negative)	52/616	12/94	40/522	0.164
Multifocality (present vs absent)	112/556	14/92	98/464	0.323
CVH (with vs without)	151/517	23/83	128/434	0.460
Bladder cancer status				
No	572 (85.6)	98 (92.5)	474 (84.3)	0.064
Previous	22 (3.3)	2 (1.9)	20 (3.6)	
Concomitant	74 (11.1)	6 (5.7)	68 (12.1)	
Tumor architecture (sessile vs papillary)	460/208	76/30	384/178	0.568
Adjuvant therapy (yes/no)	281/387	44/62	237/325	0.915

CVH, concomitant variant histology; LDH, lactate dehydrogenase; LVI, lymphovascular invasion; RNU, radical nephroureterectomy.

**Figure 1:**
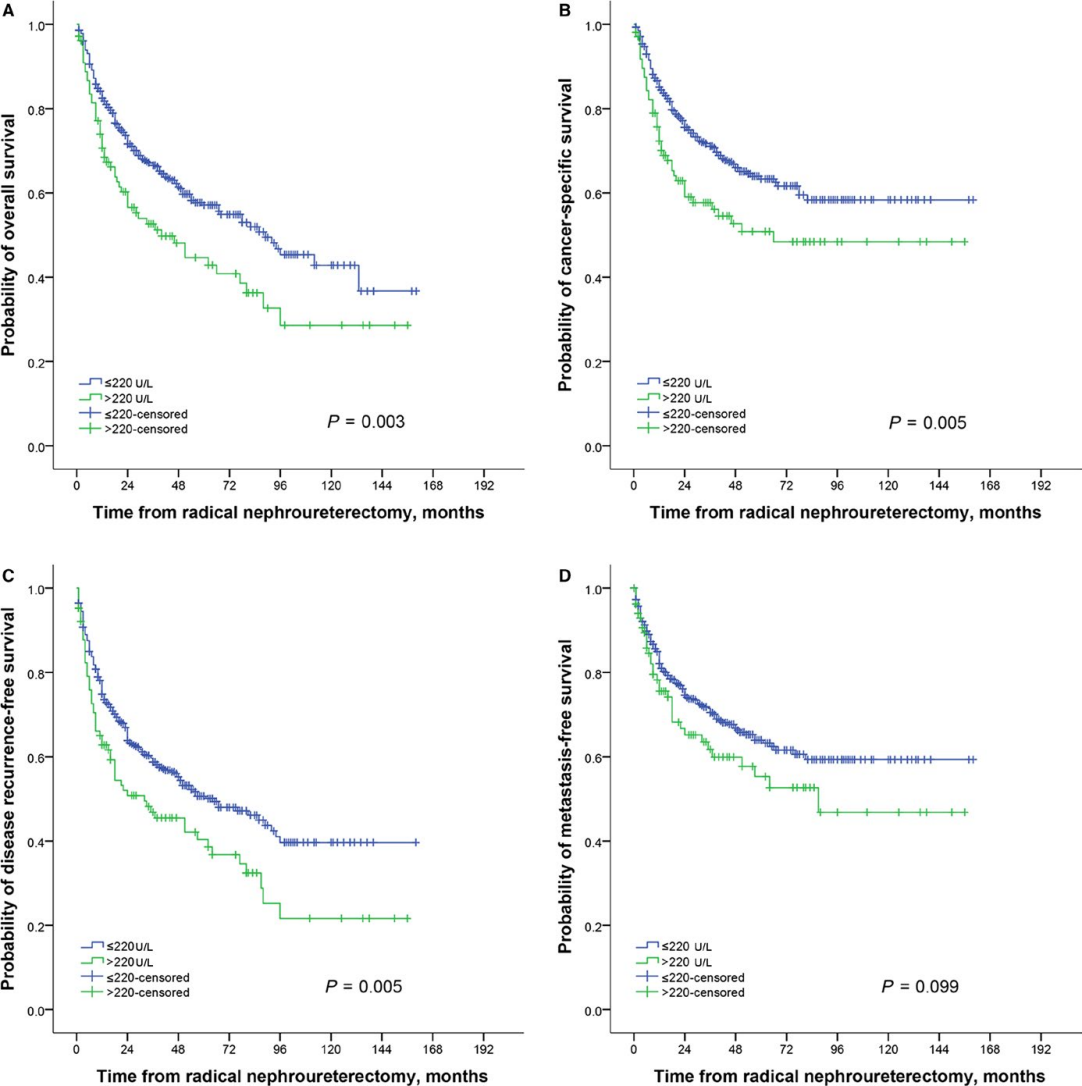
The association of LDH with the survival of UTUC patients; Overall survival (A), Cancer‐specific survival (B), Disease recurrence‐free survival (C), and Metastasis‐free survival (D)

At the last follow‐up, 243 patients (36.3%) had died from all causes and 194 (29.0%) patients had died from UTUC, respectively. The 2‐year OS and 5‐year OS were 69.2% and 55.2%, respectively. The CSS at the second and fifth year was 72.9% and 61.3%, respectively. Kaplan‐Meier plots showed the group with high serum LDH level had significant poor OS (*P* = 0.003), CSS (*P* = 0.005), and RFS (*P* = 0.005) compared with that in the group with normal serum LDH values (Figure 1A‐C), but not with respect to MFS (*P* = 0.099) (Figure 1D). Univariate Cox analysis showed that high preoperative serum LDH level was a poor prognostic factor for CSS (HR 1.54, 95%CI: 1.00‐2.38), OS (HR 1.48, 95%CI: 1.00‐2.18), RFS (HR 1.60, 95%CI: 1.12‐2.29), and MFS (HR 1.63, 95%CI: 1.04‐2.54) (Table 2). Also alkaline phosphatase, white blood cells, and globulin were significantly correlated with CSS, OS, RFS, and MFS. Albumin was only associated with CSS and OS. After controlling for the effects of standard clinicopathological features, multivariate Cox analysis showed that serum LDH value was no longer an independent predictor for CSS (HR 1.50, 95%CI: 0.87‐2.59), OS (HR 1.56, 95%CI: 0.94‐2.58), RFS (HR 1.33, 95%CI: 0.83‐2.12), or MFS (HR 1.16, 95%CI: 0.79‐1.71) (Table 3). Moreover, albumin, globulin, and HBDH were also not related to survival outcomes of UTUC patients. However, the higher level of alkaline phosphatase, anemia, tumor architecture, and CVH was shown as independent predictors for CSS and OS. In addition, results showed that tumor stage and grade, tumor size, and transfusion were significant prognostic factors for CSS, OS, RFS, and MFS. Tumor site and adjuvant therapy were also independent predictors for MFS (Table 3).

**Table 2:** Univariate analysis of survival outcomes in whole cohort

Variables	CSS			OS			RFS			MFS		
	HR	95%CI	*P*	HR	95%CI	*P*	HR	95%CI	*P*	HR	95%CI	*P*
Age (>67 vs ≤67 y)	0.93	0.70‐1.23	0.598	1.01	0.78‐1.30	0.958	0.92	0.73‐1.16	0.462	0.88	0.66‐1.18	0.39
Sex (male vs female)	0.86	0.65‐1.14	0.286	0.94	0.73‐1.21	0.641	0.92	0.73‐1.16	0.486	0.88	0.66‐1.17	0.378
Tumor site			0.723			0.815			0.671			0.190
Ureteric vs pelvic	1.01	0.73‐1.40	0.972	0.94	0.70‐1.26	0.665	0.94	0.71‐1.23	0.638	1.12	0.80‐1.57	0.499
Both vs pelvic	1.16	0.80‐1.70	0.437	1.06	0.75‐1.50	0.741	1.10	0.80‐1.52	0.555	1.42	0.97‐2.08	0.069
Tumor grade (high vs low)	3.73	2.35‐5.93	<0.001	2.95	2.03‐4.31	<0.001	2.28	1.65‐3.13	<0.001	2.51	1.67‐3.79	<0.001
LVI (with vs without)	2.68	1.94‐3.71	<0.001	2.49	1.85‐3.34	<0.001	2.19	1.65‐2.90	<0.001	2.35	1.66‐3.32	<0.001
CVH (with vs without)	2.32	1.72‐3.12	<0.001	2.09	1.60‐2.74	<0.001	1.92	1.49‐2.48	<0.001	2.08	1.53‐2.85	<0.001
Tumor size (>3 vs ≤3 cm)	2.08	1.49‐2.89	<0.001	2.04	1.52‐2.74	<0.001	1.91	1.46‐2.50	<0.001	2.00	1.43‐2.81	<0.001
Tumor architecture (sessile vs papillary)	3.81	2.52‐5.76	<0.001	2.95	2.10‐4.12	<0.001	2.47	1.84‐3.32	<0.001	2.97	2.02‐4.37	<0.001
Multifocality (yes vs no)	1.02	0.70‐1.48	0.937	0.93	0.66‐1.31	0.676	0.95	0.69‐1.31	0.765	1.14	0.79‐1.66	0.483
Surgical margin status (positive vs negative)	2.32	1.51‐3.55	<0.001	2.16	1.45‐3.20	<0.001	1.86	1.27‐2.73	0.002	1.83	1.14‐2.95	0.013
Anemia (yes vs no)	1.99	1.50‐2.63	<0.001	1.96	1.52‐2.52	<0.001	1.63	1.29‐2.07	<0.001	1.61	1.20‐2.15	0.001
Perioperative blood transfusion (yes vs no)	2.46	1.76‐3.43	<0.001	2.15	1.58‐2.93	<0.001	1.90	1.41‐2.57	<0.001	1.97	1.36‐2.85	<0.001
pT stage			<0.001			<0.001			<0.001			<0.001
pT2 vs pTis, Ta, T1	1.80	1.03‐3.14	0.038	1.68	1.05‐2.70	0.032	1.53	1.01‐2.32	0.043	2.17	1.24‐3.80	0.007
pT3 vs pTis, Ta, T1	4.06	2.56‐6.44	<0.001	3.55	2.40‐5.25	<0.001	2.98	2.12‐4.21	<0.001	4.70	2.92‐7.57	<0.001
pT4 vs pTis, Ta, T1	10.22	6.30‐16.59	<0.001	8.38	5.52‐12.74	<0.001	7.06	4.84‐10.29	<0.001	9.78	5.81‐16.45	<0.001
Lymph node status			<0.001			<0.001			<0.001			<0.001
pNx vs pN0	1.48	0.88‐2.50	0.137	1.46	0.94‐2.28	0.094	1.47	0.98‐2.21	0.065	1.45	0.86‐2.44	0.164
pN+ vs pN0	6.06	3.41‐10.78	<0.001	5.15	3.09‐8.58	<0.001	5.36	3.33‐8.62	<0.001	6.60	3.69‐11.82	<0.001
LDH (>220 U/L vs ≤220 U/L)	1.61	1.15‐2.26	0.006	1.57	1.16‐2.13	0.003	1.50	1.12‐2.00	0.006	1.36	0.94‐1.97	0.102
HBDH (>180 U/L vs ≤180 U/L)	1.38	0.95‐2.00	0.090	1.35	0.97‐1.88	0.076	1.34	0.98‐1.83	0.067	1.30	0.88‐1.93	0.188
Alkaline phosphatase (>90 vs ≤90 U/L)	1.80	1.35‐2.40	<0.001	1.51	1.16‐1.96	0.002	1.42	1.11‐1.81	0.006	1.55	1.14‐2.10	0.005
Albumin (>35 vs ≤35 g/L)	1.68	1.18‐2.39	0.004	1.69	1.23‐2.33	0.001	1.36	0.99‐1.85	0.055	1.34	0.91‐1.97	0.138
Globulin (>30 vs ≤30 g/L)	2.23	1.68‐2.96	<0.001	1.78	1.38‐2.30	<0.001	1.63	1.28‐2.07	<0.001	1.70	1.27‐2.28	<0.001
White blood cells (>8.3 vs ≤8.3*10^9^/L)	2.03	1.49‐2.76	<0.001	1.74	1.31‐2.32	<0.001	1.60	1.22‐2.10	0.001	1.48	1.05‐2.08	0.026
Adjuvant therapy (yes vs no)	0.92	0.69‐1.22	0.567	0.86	0.67‐1.11	0.253	1.10	0.87‐1.39	0.437	1.57	1.17‐2.10	0.003

CSS, cancer‐specific survival; CVH, concomitant variant histology; HBDH, alpha‐Hydroxybutyrate dehydrogenase; LDH, lactate dehydrogenase; LVI, lymphovascular invasion; MFS, metastasis‐free survival; OS, overall survival; RNU, radical nephroureterectomy; RFS, disease recurrence‐free survival.

**Table 3:** Multivariate analysis of survival outcomes in whole cohort

Variables	CSS			OS			RFS			MFS		
	HR	95%CI	*P*	HR	95%CI	*P*	HR	95%CI	*P*	HR	95%CI	*P*
Tumor site												0.005
Ureteric vs pelvic		—			—			—		1.57	1.07‐2.29	0.021
Both vs pelvic		—			—			—		1.88	1.24‐2.83	0.003
Tumor grade (high vs low)	1.90	1.15‐3.14	0.012	1.70	1.12‐2.56	0.012	1.43	1.01‐2.04	0.043	1.46	0.93‐2.31	0.102
CVH (with vs without)	1.32	0.95‐1.84	0.102	1.29	0.96‐1.74	0.095	1.19	0.90‐1.58	0.22	1.23	0.87‐1.74	0.248
LVI (with vs without)	1.12	0.77‐1.62	0.563	1.13	0.81‐1.59	0.469	0.97	0.70‐1.35	0.875	1.00	0.67‐1.48	0.991
Tumor size (>3 vs ≤3 cm)	1.67	1.17‐2.38	0.005	1.73	1.26‐2.37	0.001	1.63	1.23‐2.17	0.001	1.77	1.22‐2.56	0.003
Tumor architecture (sessile vs papillary)	1.70	1.06‐2.73	0.028	1.44	0.98‐2.13	0.067	1.39	0.98‐1.95	0.062	1.45	0.93‐2.27	0.103
Surgical margin status (positive vs negative)	1.01	0.64‐1.60	0.961	1.02	0.67‐1.55	0.931	0.94	0.62‐1.41	0.750	0.79	0.46‐1.33	0.370
Perioperative blood transfusion (yes vs no)	1.76	1.21‐2.57	0.003	1.58	1.12‐2.24	0.009	1.47	1.05‐2.05	0.024	1.58	1.04‐2.40	0.032
Anemia (yes vs no)	1.37	1.00‐1.88	0.051	1.42	1.07‐1.88	0.014	1.22	0.94‐1.59	0.13	1.20	0.87‐1.66	0.260
pT stage			0.003			<0.001			<0.001			<0.001
pT2 vs pTis, Ta, T1	1.28	0.72‐2.29	0.404	1.31	0.80‐2.13	0.289	1.26	0.82‐1.93	0.303	1.38	0.76‐2.49	0.294
pT3 vs pTis, Ta, T1	2.06	1.22‐3.47	0.007	2.03	1.30‐3.17	0.002	1.98	1.34‐2.92	0.001	2.62	1.53‐4.51	<0.001
pT4 vs pTis, Ta, T1	2.84	1.53‐5.27	0.001	2.89	1.69‐4.92	<0.001	2.99	1.83‐4.87	<0.001	3.74	1.95‐7.19	<0.001
Lymph node status			0.006			0.006			<0.001			<0.001
pNx vs pN0	2.06	1.21‐3.51	0.008	1.90	1.20‐3.00	0.006	1.95	1.28‐2.97	0.002	1.90	1.11‐3.26	0.02
pN+ vs pN0	2.77	1.47‐5.23	0.002	2.44	1.39‐4.28	0.002	2.97	1.75‐5.03	<0.001	3.76	1.99‐7.08	<0.001
LDH (>220 U/L vs ≤220 U/L)	1.35	0.77‐2.36	0.292	1.44	0.87‐2.41	0.160	1.24	0.77‐1.98	0.377	1.08	0.73‐1.60	0.691
HBDH (>180 U/L vs ≤180 U/L)	0.83	0.46‐1.52	0.552	0.81	0.47‐1.40	0.445	0.90	0.54‐1.48	0.670		‐	
Alkaline phosphatase (>90 vs ≤90 U/L)	1.63	1.19‐2.21	0.002	1.38	1.04‐1.83	0.026	1.25	0.96‐1.63	0.104	1.32	0.95‐1.82	0.099
Albumin (>35 vs ≤35 g/L)	1.00	0.67‐1.51	0.986	1.08	0.75‐1.55	0.693	0.90	0.63‐1.28	0.563	0.97	0.62‐1.50	0.874
Globulin (>30 vs ≤30 g/L)	1.35	0.99‐1.85	0.057	1.15	0.87‐1.53	0.325	1.15	0.89‐1.49	0.285	1.11	0.80‐1.54	0.529
White blood cells (>8.3 vs ≤8.3*10^9^/L)	1.49	1.05‐2.12	0.027	1.33	0.96‐1.83	0.085	1.44	1.06‐1.95	0.020	1.37	0.93‐2.02	0.107
Adjuvant therapy (yes vs no)		‐			‐			‐		1.54	1.14‐2.10	0.005

CSS, cancer‐specific survival; CVH, concomitant variant histology; HBDH, alpha‐Hydroxybutyrate dehydrogenase; LDH, lactate dehydrogenase; LVI, lymphovascular invasion; MFS, metastasis‐free survival; OS, overall survival; RFS, disease recurrence‐free survival; RNU, radical nephroureterectomy.

In subgroup analysis, results found higher LDH was only associated with poor OS in patients with localized disease (pT ≤ 2) (HR 4.03, 95%CI: 1.37‐11.88), but not in patients with advanced disease (pT ≥ 3) (HR 1.13, 95%CI: 0.62‐2.05) (Tables 4 and 5). Also, globulin was found to be an independent predictor of CSS (HR 2.39, 95%CI: 1.26‐4.52) and RFS (HR 1.89, 95%CI: 1.18‐3.02) in cases with localized UTUC.

**Table 4:** Multivariate analysis of survival outcomes in patients with localized UTUC (pT ≤ 2)

Variables	CSS			OS			RFS			MFS		
	HR	95%CI	*P*	HR	95%CI	*P*	HR	95%CI	*P*	HR	95%CI	*P*
Tumor site												0.003
Ureteric vs pelvic		—			—			—		3.27	1.52‐7.04	0.003
Both vs pelvic		—			—			—		3.58	1.53‐8.37	0.003
Tumor grade (high vs low)	1.67	0.81‐3.42	0.164	1.38	0.78‐2.42	0.270	1.27	0.79‐2.04	0.327	1.57	0.77‐3.18	0.212
CVH (with vs without)	1.05	0.39‐2.81	0.924	0.67	0.26‐1.75	0.417	0.67	0.30‐1.50	0.333	1.01	0.38‐2.66	0.981
LVI (with vs without)	1.50	0.40‐5.71	0.551	2.18	0.71‐6.73	0.174	1.11	0.36‐3.46	0.861	0.92	0.22‐3.94	0.911
Tumor size (>3 vs ≤3 cm)	0.75	0.40‐1.37	0.345	1.00	0.60‐1.68	0.988	1.03	0.67‐1.60	0.892	1.00	0.54‐1.87	0.999
Tumor architecture (sessile vs papillary)	2.53	1.27‐5.02	0.008	1.61	0.93‐2.79	0.091	1.34	0.85‐2.13	0.211	1.74	0.89‐3.42	0.107
Surgical margin status (positive vs negative)	1.84	0.50‐6.78	0.362	1.21	0.34‐4.22	0.770	0.97	0.29‐3.27	0.963	0.70	0.15‐3.39	0.660
Perioperative blood transfusion (yes vs no)	1.66	0.76‐3.61	0.201	1.07	0.53‐2.16	0.862	1.35	0.70‐2.61	0.379	2.22	1.01‐4.88	0.046
Anemia (yes vs no)	1.39	0.71‐2.73	0.334	1.46	0.83‐2.57	0.186	1.10	0.67‐1.82	0.703	1.28	0.65‐2.51	0.48
Lymph node status			0.029			0.010			0.002			0.005
pNx vs pN0	5.02	1.17‐21.58	0.030	3.89	1.38‐10.99	0.010	3.14	1.33‐7.38	0.009	2.51	0.73‐8.62	0.144
pN+ vs pN0	14.10	1.93‐103.07	0.009	10.90	2.07‐57.48	0.005	12.18	2.84‐52.34	0.001	17.68	3.02‐103.51	0.001
LDH (>220 U/L vs ≤220 U/L)	3.47	0.97‐12.39	0.056	4.03	1.37‐11.88	0.011	2.36	0.89‐6.27	0.085	3.16	0.93‐10.76	0.065
HBDH (>180 U/L vs ≤180 U/L)	0.96	0.25‐3.73	0.955	0.69	0.21‐2.22	0.533	1.02	0.36‐2.89	0.973	1.13	—	
Alkaline phosphatase (>90 vs ≤90 U/L)	1.34	0.70‐2.56	0.380	1.14	0.64‐2.02	0.651	1.05	0.63‐1.74	0.851	0.88	0.43‐1.78	0.719
Albumin (>35 vs ≤35 g/L)	0.43	0.14‐1.31	0.139	0.88	0.41‐1.92	0.751	0.71	0.34‐1.50	0.368	0.32	0.09‐1.13	0.078
Globulin (>30 vs ≤30 g/L)	2.39	1.26‐4.52	0.007	1.67	0.96‐2.91	0.067	1.89	1.18‐3.02	0.009	1.82	0.91‐3.65	0.093
White blood cells (>8.3 vs ≤8.3*10^9^/L)	1.48	0.72‐3.03	0.287	1.31	0.71‐2.43	0.393	0.95	0.53‐1.71	0.866	1.32	0.59‐2.97	0.503
Adjuvant therapy (yes vs no)		—			—			—		1.34	0.72‐2.49	0.361

CSS, cancer‐specific survival; CVH, concomitant variant histology; HBDH, alpha‐Hydroxybutyrate dehydrogenase; LDH, lactate dehydrogenase; LVI, lymphovascular invasion; MFS, metastasis‐free survival; OS, overall survival; RFS, disease recurrence‐free survival; RNU, radical nephroureterectomy.

**Table 5:** Multivariate analysis of survival outcomes in patients with advanced UTUC (pT ≥ 3)

Variables	CSS			OS			RFS			MFS		
	HR	95%CI	*P*	HR	95%CI	*P*	HR	95%CI	*P*	HR	95%CI	*P*
Tumor site												0.223
Ureteric vs pelvic		—			—			—		1.16	0.73‐1.84	0.528
Both vs pelvic		—			—			—		1.53	0.94‐2.47	0.085
Tumor grade (high vs low)	1.99	0.93‐4.28	0.076	2.02	1.03‐3.97	0.042	1.44	0.83‐2.50	0.198	1.38	0.73‐2.61	0.319
CVH (with vs without)	1.36	0.94‐1.97	0.098	1.41	1.01‐1.96	0.044	1.24	0.90‐1.70	0.182	1.27	0.86‐1.86	0.232
LVI (with vs without)	1.16	0.79‐1.70	0.456	1.16	0.82‐1.65	0.392	1.02	0.73‐1.43	0.922	1.05	0.69‐1.58	0.830
Tumor size (>3 vs ≤3 cm)	3.44	1.97‐5.98	<0.001	3.03	1.87‐4.91	<0.001	2.74	1.77‐4.23	<0.001	2.69	1.56‐4.63	<0.001
Tumor architecture (sessile vs papillary)	1.40	0.73‐2.69	0.316	1.67	0.90‐3.10	0.102	1.97	1.11‐3.51	0.021	1.54	0.82‐2.87	0.179
Surgical margin status (positive vs negative)	1.20	0.73‐1.95	0.476	1.21	0.78‐1.90	0.395	1.05	0.68‐1.62	0.840	0.99	0.57‐1.73	0.97
Perioperative blood transfusion (yes vs no)	1.85	1.19‐2.86	0.006	1.70	1.13‐2.56	0.011	1.53	1.02‐2.28	0.041	1.40	0.85‐2.32	0.189
Anemia (yes vs no)	1.44	1.00‐2.07	0.050	1.47	1.06‐2.04	0.023	1.34	0.99‐1.84	0.063	1.27	0.87‐1.85	0.213
Lymph node status			0.010			0.020			<0.001			<0.001
pNx vs pN0	1.54	0.85‐2.79	0.155	1.45	0.86‐2.45	0.166	1.73	1.05‐2.87	0.032	1.70	0.92‐3.15	0.093
pN+ vs pN0	2.53	1.33‐4.85	0.005	2.17	1.21‐3.91	0.010	3.13	1.77‐5.54	<0.001	3.92	1.97‐7.79	<0.001
LDH (>220 U/L vs ≤220 U/L)	1.12	0.59‐2.12	0.730	1.13	0.62‐2.05	0.683	0.94	0.54‐1.65	0.832	0.71	0.35‐1.46	0.352
HBDH (>180 U/L vs ≤180 U/L)	0.78	0.38‐1.59	0.488	0.74	0.38‐1.44	0.374	0.79	0.43‐1.47	0.460		‐	
Alkaline phosphatase (>90 vs ≤90 U/L)	1.56	1.08‐2.25	0.018	1.34	0.96‐1.89	0.087	1.29	0.93‐1.79	0.126	1.41	0.95‐2.09	0.092
Albumin (>35 vs ≤35 g/L)	1.39	0.87‐2.20	0.168	1.24	0.81‐1.89	0.331	0.97	0.63‐1.49	0.898	1.30	0.79‐2.14	0.307
Globulin (>30 vs ≤30 g/L)	1.15	0.80‐1.65	0.445	1.07	0.77‐1.48	0.700	1.01	0.74‐1.37	0.977	0.93	0.64‐1.37	0.726
White blood cells (>8.3 vs ≤8.3*10^9^/L)	1.47	0.98‐2.23	0.065	1.32	0.90‐1.93	0.157	1.82	1.25‐2.64	0.002	1.48	0.94‐2.35	0.093
Adjuvant therapy (yes vs no)		—			—			—		1.52	1.05‐2.20	0.028

CSS, cancer‐specific survival; CVH, concomitant variant histology; HBDH, alpha‐Hydroxybutyrate dehydrogenase; LDH, lactate dehydrogenase; LVI, lymphovascular invasion; MFS, metastasis‐free survival; OS, overall survival; RFS, disease recurrence‐free survival; RNU, radical nephroureterectomy.

## 4 DISCUSSION

Previous studies have demonstrated that high serum LDH level was associated with prognosis in several malignancies, such as renal cell carcinoma, melanoma, prostate cancer, squamous cell cancer, nasopharyngeal and colorectal cancer.^5^, ^7^ Limited evidence had shown high serum LDH level was related to unfavorable prognosis in patients with UTUC.^6^ In our study, the results suggested that high LDH level was associated with gender, perioperative blood transfusion, and tumor grade, which was also partially in agreement with that of previous published study showing that the high LDH level might reflect heavier tumor burden in gastric cancer.^6^, ^8^ Although many studies found LDH was a good prognostic predictor for cancer patients, the exact mechanism was still unclear. Lactate dehydrogenase, which regulated by hypoxia‐inducible factor‐1 alpha (HIF‐1a), is involved in the glycolytic pathway and catalyzes the conversion of pyruvate and lactate coupled with the conversion of NADH and NAD+. Thus, high LDH level could reflect the oncogenic aerobic glycolysis or the Warburg effect which can promote the malignant transformation and survival of cancer cells.^3^, ^6^, ^9^

However, the results of our cohort suggested that high serum LDH level was not associated with overall CSS, OS, RFS, or MFS in UTUC patients, which was consistent with the results reported by Kluth et al.^10^ Also, subgroup analysis in this study showed that serum LDH level could not affect survival outcomes of patients with the low‐grade disease or high‐grade UTUC. But interestingly, we found elevated serum LDH contributed to poor OS in patients with localized UTUC, which was totally different from the result of Zhang et al who reported elevated LDH was correlated with worse OS in patients having advanced disease. Although the cutoff value of LDH in Zhang et al^5^, ^6^ was set as 245 U/L, a little higher than that in our study, a recent meta‐analysis found different LDH cutoff value did not affect the HR for survival outcomes. More importantly, 100 UTUC patients with only 10 cases having high LDH level were included in the study conducted by Zhang et al^6^ and also they did not adjust other confounder biomarkers. These limitations significantly reduced the significance of their results.

In our cohort, we also found albumin, globulin, and HBDH were not associated with prognosis of UTUC patients, while higher alkaline phosphatase was found to be associated with worse CSS and OS in all patients and higher globulin was associated with poor CSS and RFS in cases with localized disease separately. In contrast, Kluth et al reported lower serum albumin level contributed to higher mortality after disease recurrence and Sheth et al found lower albumin was associated with worse RFS and OS without adjusting the impact of other confounder biomarkers.^10^, ^11^ Moreover, they did not exclude patients with liver diseases which could affect albumin levels and other biochemical indexes such as globulin, aspartate aminotransferase, and bilirubin.^10^ Previous evidence found increased alkaline phosphatase level in patients with kidney disease and liver cancer.^12^, ^13^ Kluth et al reported alkaline phosphatase was not a prognostic predictor in patients with recurrent disease, but another study including patients with high‐grade disease reported ALP ≥ 116 U/L was associated with adverse RFS and OS in univariate analysis and an AA score based on the cumulative number of alterations in albumin and alkaline phosphatase was proved to be an independent predictor of RFS and OS in multivariate analysis.^10^, ^11^

Sheth et al also suggested white blood cells were not a prognostic predictor in high‐grade patients, while our cohort found higher white blood cells to be associated with worse CSS and RFS, which was in line with Kluth et al reported higher white blood cells were correlated with a higher after disease recurrence.^10^, ^11^ Recent studies also figured out that white blood cells were associated with worse CSS in patients with bladder cancer in univariate analysis.^14^

In our study, the LVI was detected in only 99 (14.8%) patients, which was significantly lower than the majority of published studies.^15^ Like Rink et al and some other previous studies reported, we also found the presence of LVI was not a risk factor for oncologic outcomes.^16^, ^17^, ^18^, ^19^ In addition, previous studies proved that LVI was not linked to oncologic outcomes in patients already had LN metastases.^20^ As lymphadenectomy was a level C evidence recommended by guideline, it was not routinely performed in our cohort.^1^ 78.4% of patients were not staged with a lymph node dissection in our study. Thus, the prognostic impact of LVI may be underestimated in our cohort. Our results also showed that the surgical margin status was not an independent prognostic factor. Similarly, Kim et al^21^ also found the margin status was not associated with disease‐free survival or CSS in UTUC. Although the EAU guideline suggested that positive surgical margin (PSM) was an independent predictor of disease recurrence, we noticed the reference the guideline cited reported that PSM was only related to MFS but not associated with CSS or RFS.^22^ In addition, some studies found the prognostic value of surgical margin only in the univariate analysis.^22^ Moreover, Colin et al^22^ also explained that in organ‐confined disease (≤pT2), the most of PSM was caused by surgical mistake instead of the high invasive ability of cancer due to the dissection too close to the ureter when performing distal ureterectomy. Thus, the findings of surgical margins at tumor site, especially at the ureteral location, should be explained with caution. In our study, 10 cases with PSM were at a ≤pT2 stage. At last, many clinicopathological parameters were included in our multivariate models, so other factors may overpower the PSM effect.

Some limitations of this study should be mentioned. First, the retrospective nature of this study may cause a selection bias. In addition, as there is no consensus on the lymphadenectomy pattern for UTUC and the benefits of lymphadenectomy remain uncertain, lymphadenectomy was not routinely conducted and the extent of lymph node dissection was not standardized. Moreover, the data of smoking were not available in this study; however, one most recent systematic review study found that smoking was not associated with OS in UTUC patients.^23^ Finally, our cohort only included patients from our single center, and the results should be validated by well‐designed prospective multi‐institution studies in the future.

## 5 CONCLUSION

Our study has found that preoperative LDH, albumin, globulin, and HBDH were not associated with survival outcomes in patients with UTUC, although higher LDH was found to be associated with poor OS in cases with localized disease. Moreover, higher alkaline phosphatase was proved to be independently correlated with worse CSS and OS, and also higher white blood cells were an independent predictor for CSS and RFS.

## ETHICAL APPROVAL

All procedures performed in studies involving human participants were in accordance with the ethical standards of the institutional and/or national research committee and with the 1964 Helsinki declaration and its later amendments or comparable ethical standards. For this type of study, formal consent is not required.

## CONFLICT OF INTERESTS

All authors declare no conflict of interests.
